# Immunohistochemistry for c‐myc and bcl‐2 overexpression improves risk stratification in primary central nervous system lymphoma

**DOI:** 10.1002/hon.2727

**Published:** 2020-03-07

**Authors:** Stefan Hatzl, Florian Posch, Alexander Deutsch, Christine Beham‐Schmid, Herbert Stöger, Hildegard Greinix, Martin Pichler, Peter Neumeister, Katharina T. Prochazka

**Affiliations:** ^1^ Division of Hematology, Department of Internal Medicine Medical University of Graz (MUG) Austria; ^2^ Division of Oncology, Department of Internal Medicine Medical University of Graz (MUG) Austria; ^3^ Center for Biomarker Research in Medicine (CBmed Ges.m.b.H.) Austria; ^4^ Institute of Pathology, Medical University of Graz (MUG) Austria; ^5^ Research Unit for non‐coding RNAs and genome editing in cancer Medical University of Graz Austria; ^6^ Department of Experimental Therapeutics The University of Texas MD Anderson Cancer Center Houston USA USA

**Keywords:** bcl‐2, c‐myc, double expressor lymphoma, PCNSL, primary CNS lymphoma

## Abstract

Overexpression of bcl‐2 and c‐myc are defining features of double‐expressor‐lymphoma (DEL) but may also occur separately in patients with primary central nervous system lymphoma (PCNSL). Despite all progress in optimizing treatment regimen, there is lack of sufficient risk stratification models. Here, we first describe the relationship between DEL biology, the National Comprehensive Cancer Network International Prognostic Index (NCCN‐IPI), treatment response, disease progression, and mortality in PCNSL. In this study, we determined c‐myc and bcl‐2 status immunohistochemically in samples of 48 patients with newly diagnosed PCNSL and followed these patients for a median interval of 6.2 years. Twelve, 18, and 17 patients harbored none, one, or both DEL features. Corresponding overall response rates after first‐line therapy were strongly associated with DEL biology (100%, 42%, and 44% in patients with 0, 1, or 2 DEL features). Patients with one or both DEL features had a 5‐fold and 13‐fold higher 5‐year risk of progression and/or death than patients without DEL features. These associations prevailed after adjusting for the NCCN‐IPI. DEL improved the discriminatory capability of the NCCN‐IPI (*P* = .0001). Furthermore, we could show that addition of DEL biology to the NCCN‐IPI significantly improved the score's discriminatory potential both toward progression‐free survival (increase in Harell's *c* = 0.15, *P* = .005) and overall survival (increase in Harell's *c* = 0.11, *P* = .029). In conclusion, DEL biology is a strong and simple‐to‐use predictor of adverse outcome in PCNSL. Addition of DEL to the NCCN‐IPI improves its prognostic potential. Disease progression from PCNSL harboring both DEL features is invariably fatal. This defines a novel PCNSL patient subset with a great unmet need for improved therapy.

## 1 INTRODUCTION

Primary central nervous system lymphoma (PCNSL) is an aggressive and rare extranodal subtype of diffuse large B‐cell lymphoma (DLBCL) representing approximately 3% of all newly diagnosed brain tumors.^1^ Despite intensive treatment regimen including stem cell transplantation, the prognosis of patients with PCNSL is still dismal with the majority succumbing to relapse and ultimate chemoresistant disease.^2^

PCNSL displays a heterogenous disorder, both in clinical presentation and outcome, reflecting its molecular and genetic heterogeneity. Hence, stratification of patients according to their risk of disease progression, death, and thus to assign the appropriate treatment modality is challenging.^3^

Clinicians often consider prognostic scores that summarize clinical risk factors to predict the risk for disease progression, relapse, and death of patients with PCNSL in order to adjust intensity of treatment. In DLBCL, an advanced scoring model (National Comprehensive Cancer Network International Prognostic Index [NCCN‐IPI]) was developed for this purpose based on data from patients treated with rituximab, cyclophosphamide, doxorubicin, vincristine, and prednisolone.^4^ This score emphasizes the impact of older age and highly increased LDH levels, and also underscores the relevance of extranodal disease for prognosis but is not specific for central nervous system (CNS) manifestation. Besides the NCCN‐IPI, more specific prognostic models for PCNSL like the Memorial Sloan‐Kettering Cancer Center prognostic model (MSKCC‐score)^5^ and the Prognostic scoring system for primary CNS lymphomas of the International Extranodal Lymphoma Study Group (IELSG‐score)^6^ have been published. However, these scores do not include easily available prognostic markers. Over the years, immunohistochemical markers have been established to discriminate individual DLBCL subgroups and several of them—CD10, BCL‐6, MUM1, BCL‐2, and CYCLIN D1—have been associated with clinical outcome. Of note, the combination of CD10, MUM1, and BCL‐6 can divide DLBCL in germinal center (GCB‐DLBCL) and activated B center (ABC) DLBCL with about 80% concordance with the gene expression profile.^7^ However, the lack of standardization of various IHC algorithms for DLBCL cell of origin classification renders them inappropriate as reliable methods used for clinical decision making.^8^

Recently, the co‐occurrence of MYC rearrangements and either BCL2 or BCL6 was defined as double‐hit B‐cell lymphoma (DHL)—or triple‐hit lymphoma if all three abnormalities could be observed—with a particularly poor prognosis and no established efficient treatment approach.^9^, ^10^

Similarly, the concurrent expression of MYC and BCL2 as demonstrated by immunohistochemistry, so called double‐expressor lymphoma (DEL), was also associated with inferior overall and progression‐free survival.^11^, ^12^

In this study, we aimed to quantify the still unknown prognostic impact of the immunohistochemical DEL biology in patients with newly diagnosed PCNSL. Moreover, we aimed to assess whether combining DEL biology with an established prognostic index (NCCN‐IPI) can provide superior risk stratification as compared to the NCCN‐IPI alone.

## 2 PATIENTS AND METHODS

### 2.1 Study design and endpoints

In this single‐center retrospective cohort study, we considered all consecutive patients with histologically confirmed PCNSL who were diagnosed according to the 2016 World Health Organization^13^ criteria between November 2004 and July 2018 and subsequently underwent treatment at the Division of Hematology, Medical University of Graz. Among this population of n = 53 patients, paraffin embedded (FFPE) tissue for immunohistochemical assessment of DEL features was available in n = 48 patients. Patients with other histologies than DLBCL or patients seropositive for human immunodeficiency virus were excluded. Demographic, clinical, tumor, and laboratory data were ascertained from (a) the routine in‐house electronic healthcare database, (b) tissue analysis reports from the local Institute of Pathology, and (c) electronic records of 17 hospitals within our healthcare trust serving a population of approximately 1.5 million persons. All patients underwent therapies which involved high‐dose methotrexate of at least 3 g/m^2^ combined with high‐dose cytarabine (**Table S1). After treatment, patients underwent routine follow‐up examinations every 3 months within the first year, 6 months for three years, and annually thereafter. Dates of death‐from‐any‐cause were ascertained from our in‐house electronic and written documentation and expanded by query of a datalink to the Austrian Social Security Provider Association. Co‐primary endpoints of this study were the physician‐adjudicated objective response rate (ORR), progression‐free survival (PFS), and overall survival (OS). ORR was defined as a composite of either complete or partial remission during first‐line therapy. PFS was defined as the interval from the date of diagnosis until disease progression, death‐from‐any‐cause, or censoring alive, whatever came first. OS was defined as the interval from diagnosis until death‐from‐any‐cause or censoring alive. Follow‐up time for time‐to‐event graphs was truncated at 10 years. The study was approved by the local ethic committee (No. 28‐496 ex 15/16).

### 2.2 Immunohistochemical assessment of BCL2 and c‐Myc

BCL2 and c‐Myc status were assessed as previously described.^14^ Briefly, FFPE tumor tissue was stained using the UltraVision LP HRP Polymer detection system (ThermoFisher, Fremont, California; primary antibody for BCL2: Clone 124; dilution 1:200, Dako, Glostrup, Denmark; primary antibody for c‐Myc: Clone Y69, dilution 1:200, Biocare Medical, Concord). Staining results were scored based on the algorithm of Green et al. Thus, IHC expression of BCL2 < 70% and MYC < 30% was assigned a double hit score (DHS) of 0, expression of BCL2 ≥ 70% or MYC≥30% a DHS of 1, and expression of BCL2 ≥ 70% and MYC≥30% a DHS of 2, respectively.^12^

### 2.3 Statistical analysis

All statistical analyses were performed using Stata (Windows version 15, Stata Corp., Houston, Texas). Continuous variables were reported as medians [25th‐75th percentile], and categorical variables were summarized as absolute frequencies (%). We considered DEL biology both as a continuous variable as well as a three‐level ordinal variable. Baseline characteristics between patients with and without disease progression or death during follow‐up were compared with rank‐sum tests, χ^2^‐tests, and Fisher's exact tests, as appropriate. The ORR was estimated with 95% binomial exact confidence interval, and binomial regression with a normal link function was used to identify uni and multivariable predictors of ORR. Kaplan‐Meier estimators were implemented for computing PFS and OS, and the corresponding functions were compared with log‐rank tests. Median follow‐up time was estimated with the reverse Kaplan‐Meier method.^15^ Cox models were used for uni and multivariable modeling of PFS and OS rates. The potential benefit of incorporating DEL biology in pretreatment risk stratification was quantified by comparing Harell's concordance indices of five risk indices (IPI, R‐IPI, NCCN‐IPI, MSKCC, and IELSG) with and without addition of DEL biology into a Cox model, respectively.^16^ Finally, we analyzed the relationship between DEL biology and OS in patients with progression, starting from the date of progression.

## 3 RESULTS

### 3.1 Cohort description and DEL biology

Forty‐eight patients were included in the analysis (Table 1). The median age at diagnosis was 60 years [25th‐75th percentile: 53‐69], and the ABC phenotype was present in 39 (93%) of patients. We observed a high prevalence of DEL features, including 18 patients (38%) with one and 18 patients (38%) with two DEL features, respectively.

**Table 1:** Baseline characteristics of patients with PCNSL (n = 48)

Variable	n (%miss.)	Overall (n = 48)	No progression or death during follow‐up (n = 16)	Progression or death during follow‐up (n = 32)	P*
Demographics					
Female gender	48(0%)	17 (35%)	7 (44%)	10 (31%)	.393
BMI at diagnosis (kg/m^2^)	48(0%)	26.7 [23.2‐28.4]	26.1 [22.7‐29.6]	26.9 [24.0‐28.4]	.877
Age at diagnosis (years)	48 (0%)	60 [53–69]	58 [49‐68]	64 [57‐74]	.133
ECOG (points)	48 (0%)	1 [1–2]	1 [1‐2]	2 [1‐3]	.361
Tumor characteristics					
Clinical stage	48 (0%)	—	—	—	.667
I or II	—	47 (98%)	16 (100%)	31 (97%)	—
III or IV	—	1 (2%)	0 (0%)	1 (3%)	—
DLBCL “cell of origin”	42(12%)	—	—	—	.608
Activated B‐center (ABC)	—	39 (93%)	10 (91%)	29 (94%)	—
Germinal center (GC)	—	3 (7%)	1 (9%)	2 (6%)	—
DEL biology	48 (0%)	—	—	—	<.0001
0 points	—	12 (24%)	9 (56%)	3 (9%)	—
1 point	—	18 (38%)	6 (38%)	12 (38%)	—
2 points	—	18 (38%)	1 (6%)	17 (53%)	—
Involvement of deep brain structures	48 (0%)	29 (60%)	11 (69%)	18 (56%)	.404
Risk stratification systems					
IPI (points)	48 (0%)	1 [1–2]	1 [1–2]	1 [1–2]	.174
R‐IPI (points)	48 (0%)	1 [1–2]	1 [1–2]	1 [1–2]	.174
NCCN‐IPI (points)	48 (0%)	3 [3–4]	3 [2‐4]	4 [3‐4]	.098
MSKCC (points)	48 (0%)	1 [1–2]	1 [1‐2]	2 [1‐2]	.132
IELSG^a^ (points)	48 (0%)	1 [1–2]	1 [1–2]	1 [1‐2]	.638

*Note:* Distribution overall and by PFS status. Data are reported as medians [25th‐75th percentile] or as absolute counts (%). n (%miss.) reports the number of patients with fully observed data for the respective variable (% missing). *P‐values are either from rank‐sum tests, χ^2^‐tests, or Fisher's exact tests, as appropriate.

Abbreviations: BMI, body mass index; DEL, double expressor lymphoma; DLBCL, diffuse large B‐cell lymphoma; ECOG, Eastern Cooperative Oncology Group performance status; IELSG, International Extranodal Lymphoma Study Group risk score for PCNSL outcomes (without cerebrospinal fluid); IPI, International Prognostic Index; MSKCC, Memorial Sloan Kettering Cancer Centre risk score for PCNSL outcomes; NCCN‐IPI, National Comprehensive Cancer Network International Prognostic Index; R‐IPI, Revised International Prognostic Index.

a The IELSG score is calculated without cerebrospinal fluid.

During treatment, 33 patients developed an objective response, including 24 complete and 9 partial remissions, for an ORR of 69% (95%CI: 54‐81), respectively. No “stable disease” responses were observed. During a median follow‐up of 6.2 years, 32 patients (66%) developed disease progression (n = 15 with primary progressive disease during treatment, n = 17 with relapse after initial treatment) and 31 patients (65%) died, for 5‐year PFS and OS estimates of 33% (95%CI: 19‐47) and 37% (22‐51), respectively (**Supplementary Figure S1). Double hit score features were highly prevalent in DLBCL patients, with more than one third of the population having one DEL feature and more than one third having two DEL features, respectively. Notably, DEL biology was not associated with any of the baseline characteristics under study (Table S2).

### 3.2 DEL biology predicts poor treatment response in PCNSL

**Figure 1:**
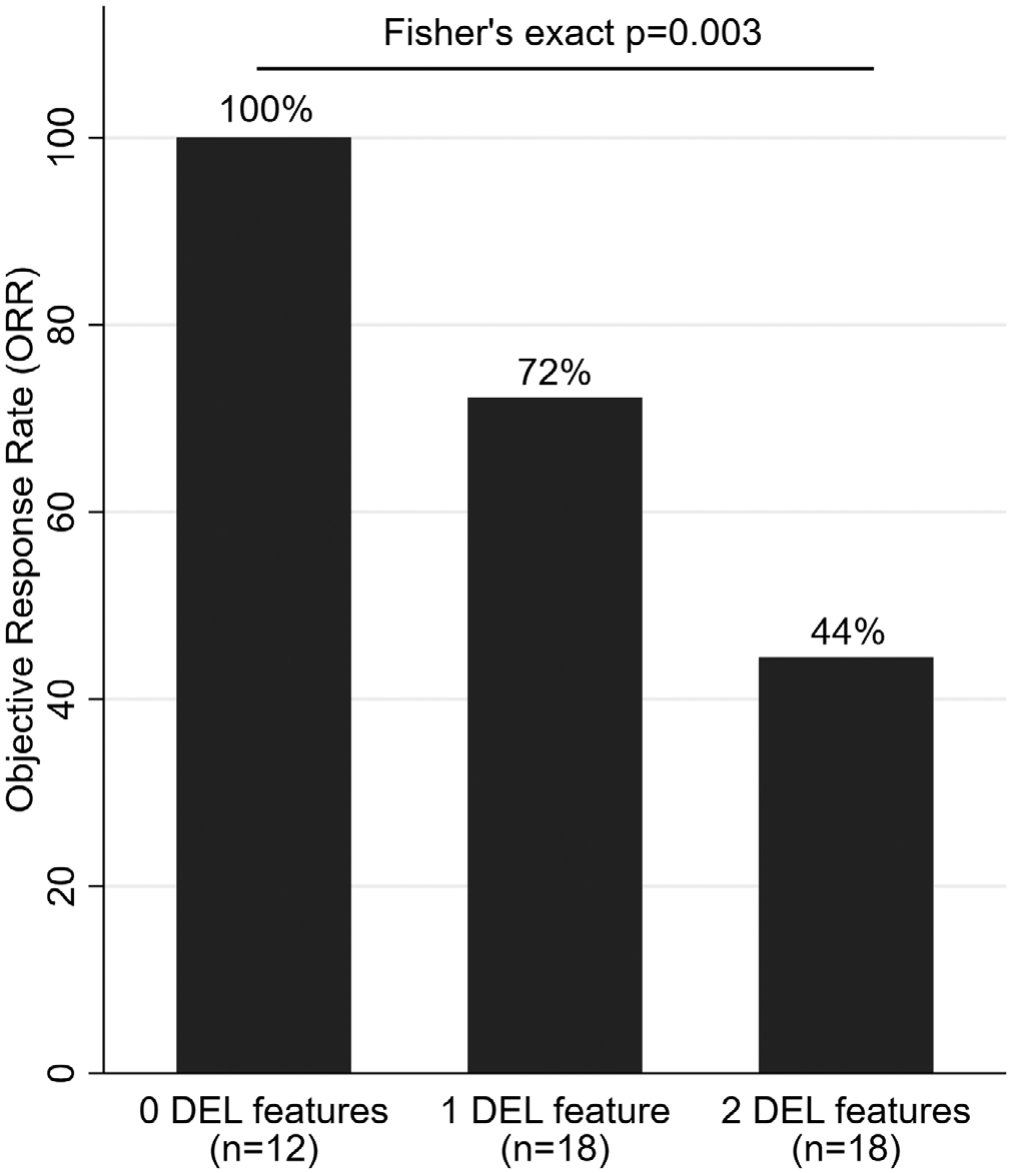
ORR to first‐line therapy in patients with PCNSL according to DEL biology (n = 48). Patients with one or both DEL feature(s) had a significantly worse ORR than patients without any DEL features in whom the ORR was 100%. Abbreviations: ORR, objective response rate; PCNSL, primary CNS lymphoma; DEL, double‐expressing lymphoma

The most frequent type of first‐line therapy was high‐dose methotrexate combined with cytarabine (n = 27, 56%). During first‐line therapy, we observed 24 complete remissions, 9 partial remissions, and 15 primary refractory disease progressions, for an ORR of 69% (95%CI: 54‐81). No stable disease outcomes were observed. Double expressor biology was strongly associated with a lower response rate (Fisher's exact *P* = .003, Figure 1). In univariable generalized linear regression analysis of response rate, the only predictors of lower ORR were DEL biology, higher age, and higher NCCN‐IPI scores (Table S3). The association between DEL biology and worse ORR prevailed upon multivariable adjustment for the NCCN‐IPI (Adjusted change in the ORR per 1 DEL feature increase = −26%, 95%CI: −35‐[−16], *P* < .0001).

### 3.3 DEL biology strongly associates with poor PFS and OS in PCNSL

**Figure 2:**
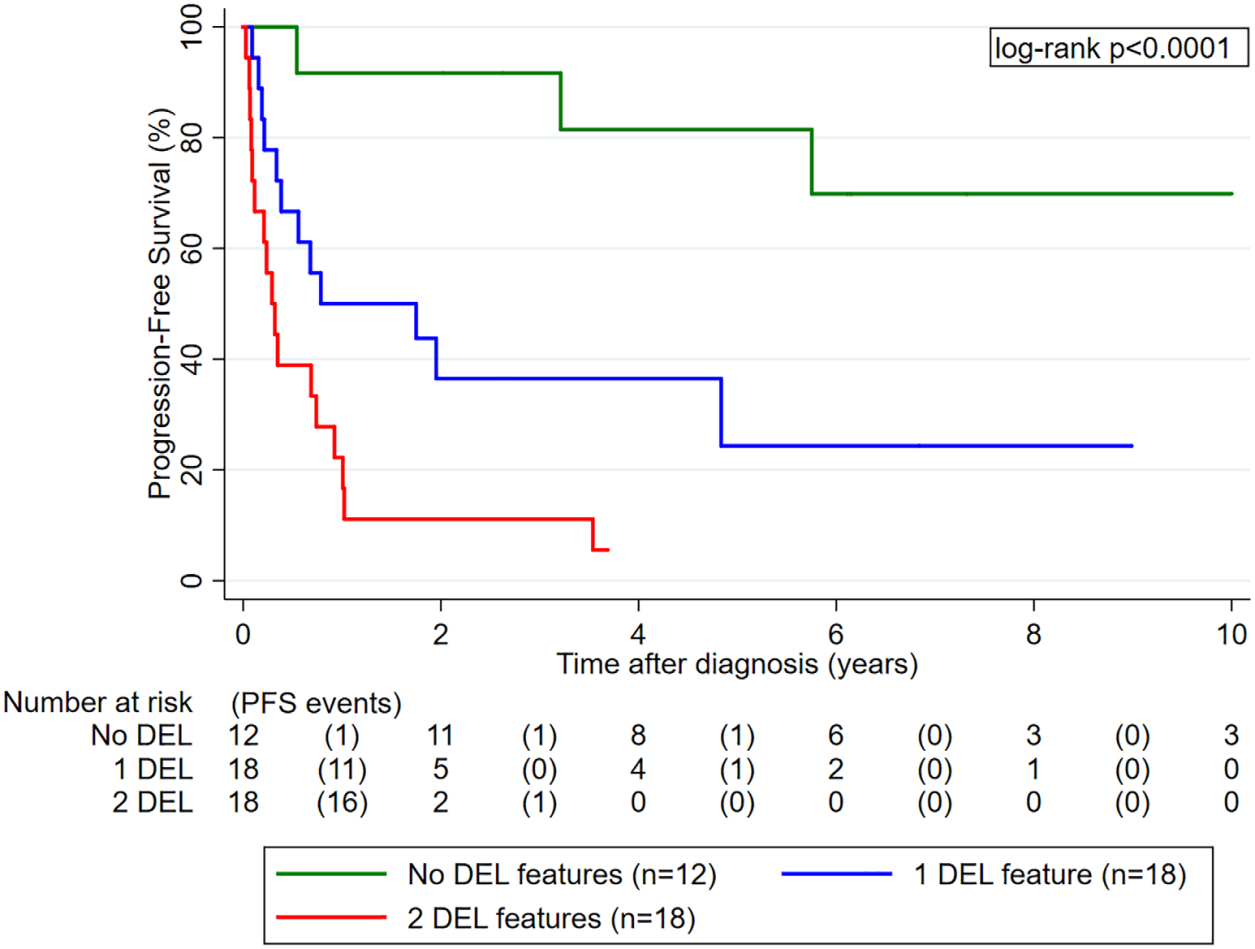
PFS experience of PCNSL patients according to DEL biology (n = 48). Patients with one or both DEL feature(s) had a significantly worse PFS than patients without any DEL features. Abbreviations: PFS, progression‐free survival; PCNSL, primary CNS lymphoma; DEL, double‐expressing lymphoma

DEL biology strongly predicted for poor PFS. In detail, median PFS was (1) not reached in patients without DEL features, (2) 0.8 years (0.3‐not reached) in patients with one DEL feature, and (3) 0.3 years (0.1‐0.7) in patients with two DEL features, respectively (log‐rank *P* < .0001, Figure 2). In univariable time‐to‐PFS regression, patients with 1 and 2 DEL features experienced 5‐fold and 13‐fold higher risks of progression and/or death than patients with 0 DEL features, respectively. Other univariable predictors of poor PFS were higher age, and the NCCN‐IPI (Table S4). The prognostic association between DEL biology and worse PFS was independent of the NCCN‐IPI (Table S5).

**Figure 3:**
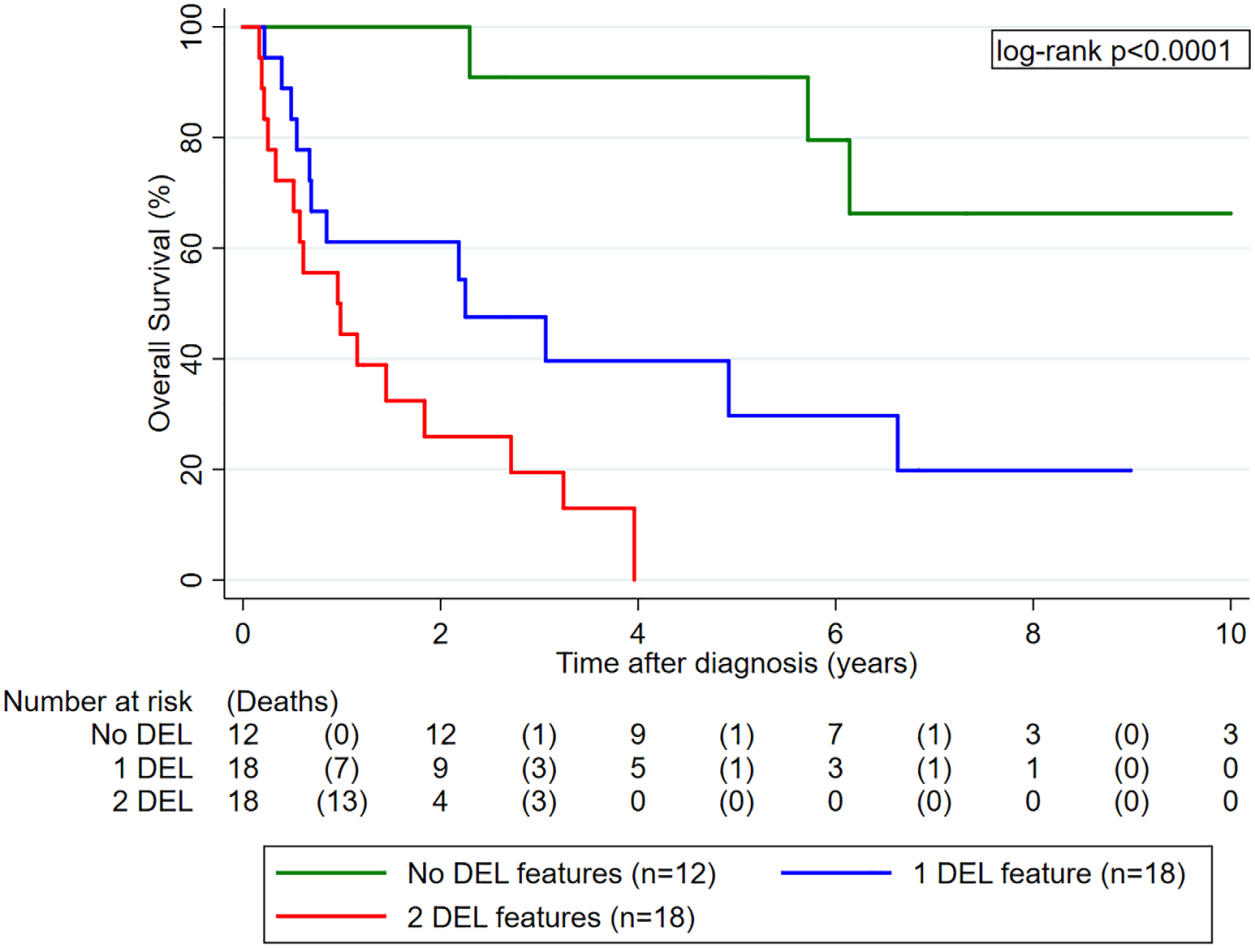
OS experience of PCNSL patients according to DEL biology (n = 48). Patients with one or both DEL feature(s) had a significantly worse OS than patients without any DEL features. Abbreviations: OS, overall survival; PCNSL, primary CNS lymphoma; DEL, double‐expressing lymphoma

Similarly, OS was significantly worse in patients with DEL biology. In detail, median OS estimates were not reached, 2.3 years (0.7‐6.6), and 1.0 years (0.3‐1.8) in patients with 0, 1, and 2 DEL features, respectively (log‐rank *P* < .0001, Figure 3). In univariable Cox regression, higher age, higher IPI, higher R‐IPI, higher NCCN‐IPI, and higher MSKCC score emerged as additional predictors of worse OS (Table S4). The association between DEL biology and poor OS prevailed upon adjusting for the NCCN‐IPI, and the NCCN‐IPI remained statistically significantly associated with poor OS also independently of DEL biology (Table S5).

### 3.4 DEL biology improves the discriminatory potential of the NCCN‐IPI toward PFS and OS in PCNSL

Among five risk assessment models for PFS and OS (IPI, R‐IPI, NCCN‐IPI, MSKCC, and IELSG), only the NCCN‐IPI showed discrimination that was consistently better than chance (ie, lower confidence band of Harell's between patients who did and did not develop disease progression and/or died above 0.5, Table 2). In contrast, DEL biology alone featured high *c*‐indices for both PFS and OS. Addition of DEL biology to the NCCN‐IPI significantly improved the score's discriminatory potential both toward PFS (Increase in Harell's *c* = 0.15, 95%CI: 0.05‐0.24, *P* = .005) and OS (Increase in Harell's *c* = 0.11, 95%CI: 0.01‐0.20, *P* = .029), respectively.

**Table 2:** Harell's *c*‐indices for five risk scores, DEL biology, and the combination of the NCCN‐IPI and DEL biology

	PFS		OS	
Risk model	Harell's *c*	95%CI	Harell's *c*	95%CI
IPI	0.59	0.49‐0.69	0.63	0.53‐0.73
R‐IPI	0.59	0.49‐0.69	0.63	0.53‐0.73
NCCN‐IPI	0.61	0.51‐0.71	0.66	0.55‐0.76
MSKCC	0.59	0.49‐0.69	0.61	0.51–0.71
IELSG	0.54	0.45‐0.64	0.57	0.47‐0.66
DEL	0.73	0.65‐0.81	0.72	0.64‐0.80
NCCN‐IPI + DEL	0.76	0.67‐0.84	0.76	0.67‐0.85

*Note:* Data are reported for the PFS and OS endpoint separately. Harell's *c*‐index quantifies discrimination, that is, the probability that among two randomly selected patients of whom one will develop the outcome and one will not develop the outcome the one with the outcome will have a higher value of the predictor variable. Harell's *c*‐indices range from 0 to 1, with 0.5 indicating a discriminator that is not better than chance.

Abbreviations: 95%CI, 95% confidence interval; DEL, double expressor lymphoma; IELSG, International Extranodal Lymphoma Study Group risk score for PCNSL outcomes (without cerebrospinal fluid); IPI, International Prognostic Index; MSKCC, Memorial Sloan Kettering Cancer Centre risk score for PCNSL outcomes; NCCN‐IPI, National Comprehensive Cancer Network International Prognostic Index; OS, overall survival;PFS, progression‐free survival; R‐IPI, Revised International Prognostic Index.

### 3.5 Relapse of PCNSL is almost univariably fatal

Among the 32 patients who developed relapse, 3 (9%), 12 (38%), and 17 (53%) patients had zero, one, or two DEL features, respectively. Relapse of PCNSL was almost univariably fatal, with 31 (97%) of the 32 patients succumbing to their illness (all deaths were due to PCNSL progression). This corresponded to a median OS after diagnosis of relapse of 0.4 years (95%CI: 0.2‐0.7, Figure S2). DEL biology did not significantly modify the OS experience after relapse (log‐rank *P* = .432), although numerically it appeared that the small group of patients with relapse and zero DEL features may have had a slightly longer time‐to‐death than patients with one or two DEL features, respectively (Figure S3).

## 4 DISCUSSION

Despite the development of novel and intensified therapy regimens, PCNSL has a very poor prognosis, nonetheless its rising incidence.^17^ Especially the risk assessment at the time of diagnosis remains unsatisfactory although the existence of several prognostic scoring systems is given. Several studies in the last years have proven the genetic heterogeneity of DLBCL, the histologic subtype representing the majority of PCNSL.^18^ In DLBCL, the identification of different genetic subtypes has led to the development of different therapeutic strategies to overcome the dismal outcome of specific subtypes.^19^ In PCNSL, the activated B‐cell like subtype accounts for the vast majority of lymphoma, known for its inferior outcome in DLBCL.^20^ Additionally, double‐hit and double‐expressor determination defined new DLBCL subtypes, already implemented in the recent WHO classification^13^ and also showing a change in routine therapeutic regimens.^10^ Despite this knowledge for the importance of the histology, none of the currently used scoring systems incorporates this factor in their prognosis system. The IELSG scoring system is based on five parameters (age, LDH, Eastern Cooperative Oncology Group score, involvement of deep brain structures and cerebrospinal fluid [CSF] protein levels), distinguishing patients in three risk groups.^6^ In several confirmation studies, the parameter of CSF protein levels emerged to be questionable, because of the high rate of missing values for this parameter and therefore an incomplete scoring.^21^ Another widely used scoring system, the Memorial Sloan‐Kettering Cancer Centre score, using only patient age and performance status also discriminate three distinct risk groups with OS varying between 7 and 90 months.^5^ Again, confirmation studies were ambiguous about its validity. Nevertheless, both scores do not include lymphoma specific characteristics like the double‐expressor status.^22^ To our knowledge, this is the first study, investigating the role of the double expressor status in PCNSL and its prognostic impact on the outcome of patients. We could clearly demonstrate, that patients with one or two double expressor features perform statistically dismal not only concerning response to the used therapeutic regimens but furthermore show obviously poor prognosis in PFS and OS. Next to the double expressor status, we also validated the R‐IPI, the NCCN‐IPI, developed for nodal DLBCL, and the PCNSL specific IELSG and MSKCC score. We could demonstrate that the MSKCC was able to discriminate different risk groups in our cohort but not the IELSG whereas our percentage of missing data concerning the CSF was quite high and therefore we had to calculate with this parameter missing, like other authors before.^23^, ^24^, ^25^ The double expressor biology remained statistically significant and showed the highest *c*‐index, especially when combined with the NCCN‐IPI.

Nevertheless, our study has some limitations. First, selection and information bias cannot be ruled out due to the study's retrospective and monocentric design. Second, DEL status has been ascertained by immunohistochemistry. This may be an issue in the sense that immunohistochemistry expression grading is highly observer‐dependent, and the optimal immunohistochemistry cut‐offs for defining positivity are also a matter of ongoing debate, in contrast to double‐hit character. We have tried to address this critical issue by performing the immunohistochemistry assessment by a single hematopathologist with >30 years of clinical experience rather than multiple hematopathologists, and furthermore using the immunohistochemistry cut‐offs suggested by Green et al, which in our opinion represent the most stringent definition of DEL immunohistochemistry status to date.^12^ However, immunohistochemistry results may still differ from “gold‐standard” methods that investigate amplification of MYC and BCL‐2 on a genetic level, such as FISH. Therefore, external validation of our results in prospective studies in an extended cohort is desirable.

In conclusion, we could demonstrate that patients with PCNSL and DEL biology have very poor outcomes and are in urgent need of new therapeutic options. Addition of DEL biology dramatically improved the NCCN‐IPI's discriminatory potential toward clinical outcomes.

## CONFLICT OF INTEREST

None of the contributing authors has any conflicts of interest, including specific financial interests and relationships and affiliations relevant to the subject matter or materials discussed in the manuscript.

## Supporting information


**Figure S1** Progression‐free (PFS, blue line) and Overall survival (OS, red line) experience of the total study cohort (n = 48).
**Figure S** **2** Overall survival (OS, blue line) with 95% confidence bands (grey shaded are) after relapse in PCNSL patients who developed relapse during follow‐up (n = 32).
**Figure S** **3** Overall survival after relapse in PCNSL patients who developed relapse during follow‐up according to DEL biology (n = 32).
**Table S1** Tabulation of first‐line treatments and salvage treatments for PCNSL (n = 48). Data are absolute counts (column %). Abbreviations: R ‐ Rituximab; HD‐MTX ‐ high dose methotrexate with a dosage of at least 3 g/m2 body surface area; HD‐AraC ‐ high dose Cytarabine with a dosage of at least 2 g/m2 body surface area; ASCT‐ Autologous Stem Cell Transplantation; Tem ‐ Temozolomide; Dexa ‐ Dexamethasone, BSC ‐ Best Supportive Care
**Table S** **2** Baseline characteristics of patients with PCNSL according to DEL biology (n = 48). Data are reported as medians [25th‐75th percentile] or as absolute counts (%). *P‐values are either from Kruskal‐Wallis tests, χ2‐tests, or Fisher's exact tests, as appropriate. **Test for trend. Abbreviations: DEL ‐ Double expressor lymphoma, BMI ‐ Body Mass Index, ECOG ‐ Eastern Cooperative Oncology Group performance status, DLBCL ‐ Diffuse Large B‐cell lymphoma, IPI ‐ International Prognostic Index, R‐IPI ‐ Revised International Prognostic Index, NCCN‐IPI ‐ National Comprehensive Cancer Network International Prognostic Index, MSKCC ‐ Memorial Sloan Kettering Cancer Centre risk score for PCNSL outcomes, IELSG ‐ International Extranodal Lymphoma Study Group risk score for PCNSL outcomes (without cerebrospinal fluid).
**Table S** **3** Univariable predictors of the objective response rate (ORR) to first‐line therapy in patients with PCNSL (n = 48). All data are from univariable binomial regression models with a normal link function. *β is the regression coefficient, that is, the change in the probability of ORR per 1 unit increase in the predictor variable. For example, the coefficient of −0.09 for ECOG means that according to the model, one‐point increase in the ECOG is associated with a 9% decrease in the ORR. **Clinical stage is not analysed as only 1 patient had stage III/IV disease. Abbreviations: 95%CI ‐ 95% confidence interval, p ‐ Wald test P‐value, BMI ‐ Body Mass Index, ECOG ‐ Eastern Cooperative Oncology Group performance status, DLBCL ‐ Diffuse Large B‐cell lymphoma, DEL ‐ double expressor lymphoma, IPI ‐ International Prognostic Index, R‐IPI ‐ Revised International Prognostic Index, NCCN‐IPI ‐ National Comprehensive Cancer Network International Prognostic Index, MSKCC ‐ Memorial Sloan Kettering Cancer Centre risk score for PCNSL outcomes, IELSG ‐ International Extranodal Lymphoma Study Group risk score for PCNSL outcomes (without cerebrospinal fluid).
**Table S** **4** Univariable predictors of first‐line progression‐free survival (PFS) and overall survival (OS) in patients with PCNSL (n = 48). All data are from univariable Cox proportional hazards regression models. *Clinical stage was not analysed as only 1 patient had stage III/IV disease. Abbreviations: PFS ‐ Progression‐free survival, OS ‐ Overall survival, HR ‐ Hazard ratio, 95%CI ‐ 95% confidence interval, p ‐ Wald test P‐value, BMI ‐ Body Mass Index, ECOG ‐ Eastern Cooperative Oncology Group performance status, N/A ‐ not applicable, DLBCL ‐ Diffuse Large B‐cell lymphoma, DEL ‐ double expressor lymphoma, IPI ‐ International Prognostic Index, R‐IPI ‐ Revised International Prognostic Index, NCCN‐IPI ‐ National Comprehensive Cancer Network International Prognostic Index, MSKCC ‐ Memorial Sloan Kettering Cancer Centre risk score for PCNSL outcomes, IELSG ‐ International Extranodal Lymphoma Study Group risk score for PCNSL outcomes (without cerebrospinal fluid).
**Table S** **5** Two multivariable models of DEL biology and the NCCN‐IPI for first‐line progression‐free survival (PFS) and overall survival (OS) in patients with PCNSL (n = 48). All data are from multivariable Cox proportional hazards regression models. Abbreviations: 95%CI ‐ 95% confidence interval, p ‐ Wald test P‐value, PFS ‐ Progression‐free survival, OS ‐ Overall survival, DEL ‐ double expressor lymphoma, NCCN‐IPI ‐ National Comprehensive Cancer Network International Prognostic Index. Ref. ‐ Reference category.
