# Supplementary material for: Immunohistochemistry for c‐myc and bcl‐2 overexpression improves risk stratification in primary central nervous system lymphoma
Source: Hematol Oncol. 2020 Mar 7;38(3):277–83. doi: 10.1002/hon.2727 (PMC7496545; doi:10.1002/hon.2727)
Supplement: Supplementary file 1 — Figure S1 Progression‐free (PFS, blue line) and Overall survival (OS, red line) experience of the total study cohort (n = 48). Figure S 2 Overall survival (OS, blue line) with 95% confidence bands (grey shaded are) after relapse in PCNSL patients who developed relapse during follow‐up (n = 32). Figure S 3 Overall survival after relapse in PCNSL patients who developed relapse during follow‐up according to DEL biology (n = 32). Table S1 Tabulation of first‐line treatments and salvage treatments for PCNSL (n = 48). Data are absolute counts (column %). Abbreviations: R ‐ Rituximab; HD‐MTX ‐ high dose methotrexate with a dosage of at least 3 g/m2 body surface area; HD‐AraC ‐ high dose Cytarabine with a dosage of at least 2 g/m2 body surface area; ASCT‐ Autologous Stem Cell Transplantation; Tem ‐ Temozolomide; Dexa ‐ Dexamethasone, BSC ‐ Best Supportive Care Table S 2 Baseline characteristics of patients with PCNSL according to DEL biology (n = 48). Data are reported as medians [25th‐75th percentile] or as absolute counts (%). *P‐values are either from Kruskal‐Wallis tests, χ2‐tests, or Fisher's exact tests, as appropriate. **Test for trend. Abbreviations: DEL ‐ Double expressor lymphoma, BMI ‐ Body Mass Index, ECOG ‐ Eastern Cooperative Oncology Group performance status, DLBCL ‐ Diffuse Large B‐cell lymphoma, IPI ‐ International Prognostic Index, R‐IPI ‐ Revised International Prognostic Index, NCCN‐IPI ‐ National Comprehensive Cancer Network International Prognostic Index, MSKCC ‐ Memorial Sloan Kettering Cancer Centre risk score for PCNSL outcomes, IELSG ‐ International Extranodal Lymphoma Study Group risk score for PCNSL outcomes (without cerebrospinal fluid). Table S 3 Univariable predictors of the objective response rate (ORR) to first‐line therapy in patients with PCNSL (n = 48). All data are from univariable binomial regression models with a normal link function. *β is the regression coefficient, that is, the change in the probability of ORR per 1 unit increase in [file HON-38-277-s001.docx]

**Supplementary Figures**

**Supplementary Figure 1. Progression-free (PFS, blue line) and Overall survival (OS, red line) experience of the total study cohort (n=48).**

**Supplementary Figure 2. Overall survival (OS, blue line) with 95% confidence bands (grey shaded are) after relapse in PCNSL patients who developed relapse during follow-up (n=32).**

**Supplementary Figure 3. Overall survival after relapse in PCNSL patients who developed relapse during follow-up according to DEL biology (n=32).**

**Supplementary Tables**

**Supplementary Table 1. Tabulation of 1^st^-line treatments and salvage treatments for PCNSL (n=48).** Data are absolute counts (column %). Abbreviations: R – Rituximab; HD-MTX - high dose methotrexate with a dosage of at least 3g/m² body surface area; HD-AraC – high dose Cytarabine with a dosage of at least 2g/m² body surface area; ASCT– Autologous Stem Cell Transplantation; Tem – Temozolomide; Dexa – Dexamethasone, BSC - Best Supportive Care

|  |  | **No DEL feature (n=12)** | **One DEL feature (n=18)** | **Two DEL features (n=18)** | **p*** |
| --- | --- | --- | --- | --- | --- |
|  |  |  |  |  |  |
| **1^st^ line treatment** |  |  |  |  |  |
| R-HD-MTX+HD-AraC |  | 5 (42%) | 7 (39%) | 6 (33%) | 0.43 |
| MATRIX ^1^ |  | 4 (33%) | 5 (28%) | 6 (33%) | 0.57 |
| “Freiburg Protocol” ^2,3^ |  | 2 (16%) | 2 (11%) | 4 (22%) | 0.31 |
| HD-MTX+HD-AraC |  | 1 (9%) | 3 (17%) | 2 (11%) | 0.25 |
| **ASCT after 1^st^ line** |  | 7 (58%) | 9 (50%) | 11 (61%) | 0.46 |
|  |  |  |  |  |  |
| **Relapsed patients**  **(n =32)** |  | **No DEL feature (n=3)** | **One DEL feature (n=12)** | **Two DEL features (n=17)** | **p*** |
| **Salvage treatment** |  |  |  |  |  |
| Radiotherapy |  | 2 (66%) | 7 (58%) | 10 (58%) | 0.77 |
| HD-MTX |  | -- | 3 (25%) | 4 (24%) | 0.15 |
| Tem + Dexa |  | 1 (33%) | -- | 1 (6%) | 0.19 |
| BSC |  | -- | 2 (17%) | 2 (12 %) | 0.13 |

**Supplementary References for Table 1**

1. Ferreri AJ, Cwynarski K, Pulczynski E, et al. Chemoimmunotherapy with methotrexate, cytarabine, thiotepa, and rituximab (MATRix regimen) in patients with primary CNS lymphoma: Results of the first randomisation of the international extranodal lymphoma study group-32 (IELSG32) phase 2 trial. *Lancet Haematol*. 2016;3(5):e217-27.

2. Illerhaus G, Marks R, Ihorst G, et al. High-dose chemotherapy with autologous stem-cell transplantation and hyperfractionated radiotherapy as first-line treatment of primary CNS lymphoma. *J Clin Oncol*. 2006;24(24):3865-3870.

3. Illerhaus G, Muller F, Feuerhake F, Schafer AO, Ostertag C, Finke J. High-dose chemotherapy and autologous stem-cell transplantation without consolidating radiotherapy as first-line treatment for primary lymphoma of the central nervous system. *Haematologica*. 2008;93(1):147-148.

**Supplementary Table 2. Baseline characteristics of patients with PCNSL according to DEL biology (n=48).** Data are reported as medians [25^th^-75^th^ percentile] or as absolute counts (%). *p-values are either from Kruskal-Wallis tests, χ^2^-tests, or Fisher’s exact tests, as appropriate. **Test for trend. Abbreviations: DEL – Double expressor lymphoma, BMI – Body Mass Index, ECOG – Eastern Cooperative Oncology Group performance status, DLBCL – Diffuse Large B-cell lymphoma, IPI – International Prognostic Index, R-IPI – Revised International Prognostic Index, NCCN-IPI – National Comprehensive Cancer Network International Prognostic Index, MSKCC – Memorial Sloan Kettering Cancer Centre risk score for PCNSL outcomes, IELSG – International Extranodal Lymphoma Study Group risk score for PCNSL outcomes (without cerebrospinal fluid).

| **Variable** |  | **No DEL feature (n=12)** | **One DEL feature (n=18)** | **Two DEL features (n=18)** | **p*** |
| --- | --- | --- | --- | --- | --- |
|  |  |  |  |  |  |
| **Demographics** |  |  |  |  |  |
| Female gender |  | 4 (33%) | 6 (33%) | 7 (39%) | 0.999 |
| BMI at diagnosis (kg/m²) |  | 26.0 [21.4-28.0] | 26.2 [22.7-28.0] | 27.2 [25.4-29.1] | 0.454 |
| Age at diagnosis (years) |  | 60 [48-66] | 65 [54-69] | 61 [53-74] | 0.442 |
| ECOG (points) |  | 1 [1-2] | 2 [1-3] | 1 [1-2] | 0.846 |
|  |  |  |  |  |  |
| **Tumour characteristics** |  |  |  |  |  |
| Clinical stage |  | / | / | / | 0.999 |
| ---I or II |  | 12 (100%) | 18 (100%) | 17 (94%) | / |
| ---III or IV |  | 0 (0%) | 0 (0%) | 1 (6%) | / |
| DLBCL “cell of origin”** |  | / | / | / | 0.180 |
| ---Activated B-centre (ABC) |  | 5 (71%) | 18 (100%) | 16 (94%) | / |
| ---Germinal centre (GC) |  | 2 (29%) | 0 (0%) | 1 (6%) | / |
| Involvement of deep brain structures |  | 8 (67%) | 12 (67%) | 9 (50%) | 0.605 |
|  |  |  |  |  |  |
| **Risk stratification systems** |  |  |  |  |  |
| IPI (points) |  | 1 [1-2] | 2 [1-2] | 1 [1-2] | 0.709 |
| R-IPI (points) |  | 1 [1-2] | 2 [1-2] | 1 [1-2] | 0.709 |
| NCCN-IPI (points) |  | 3 [2-4] | 4 [3-4] | 4 [3-4] | 0.326 |
| MSKCC (points) |  | 1 [1-2] | 2 [1-2] | 1 [1-2] | 0.487 |
| IELSG (points) |  | 1 [1-1] | 2 [1-2] | 1 [1-2] | 0.396 |

**Supplementary Table 3. Univariable predictors of the objective response rate (ORR) to 1^st^-line therapy in patients with PCNSL (n=48).** All data are from univariable binomial regression models with a normal link function. *β is the regression coefficient, i.e. the change in the probability of ORR per 1 unit increase in the predictor variable. For example, the coefficient of -0.09 for ECOG means that according to the model, one-point increase in the ECOG is associated with a 9% decrease in the ORR. **Clinical stage is not analysed as only 1 patient had stage III/IV disease. Abbreviations: 95%CI – 95% confidence interval, p – Wald test p-value, BMI – Body Mass Index, ECOG – Eastern Cooperative Oncology Group performance status, DLBCL – Diffuse Large B-cell lymphoma, DEL – double expressor lymphoma, IPI – International Prognostic Index, R-IPI – Revised International Prognostic Index, NCCN-IPI – National Comprehensive Cancer Network International Prognostic Index, MSKCC – Memorial Sloan Kettering Cancer Centre risk score for PCNSL outcomes, IELSG – International Extranodal Lymphoma Study Group risk score for PCNSL outcomes (without cerebrospinal fluid).

| **Variable** |  | **β*** | **95%CI** | **p** |
| --- | --- | --- | --- | --- |
|  |  |  |  |  |
| **Demographics** |  |  |  |  |
| Female gender |  | -0.15 | -0.43-0.13 | 0.282 |
| BMI at diagnosis (per 5kg/m² increase) |  | -0.05 | -0.24-0.15 | 0.634 |
| Age at diagnosis (per 5 years increase) |  | -0.04 | -0.06-(-0.03) | <0.0001 |
| ECOG (per 1 point increase) |  | -0.09 | -0.22-0.03 | 0.141 |
|  |  |  |  |  |
| **Tumour characteristics** |  |  |  |  |
| Clinical stage III/IV** |  | N/A | N/A | N/A |
| DLBCL “Cell of origin” Germinal centre (GC) |  | 0.03 | -0.52-0.58 | 0.928 |
| DEL biology (per 1 DEL feature increase) |  | -0.28 | -0.38-(-0.18) | <0.0001 |
| Involvement of deep brain structures |  | 0.18 | -0.09-0.45 | 0.194 |
|  |  |  |  |  |
| **Risk stratification systems** |  |  |  |  |
| IPI (per 1 point increase) |  | -0.11 | -0.25-0.02 | 0.100 |
| R-IPI (per 1 point increase) |  | -0.11 | -0.25-0.02 | 0.100 |
| NCCN-IPI (per 1 point increase) |  | -0.13 | -0.18-(-0.08) | <0.0001 |
| MSKCC (per 1 point increase) |  | -0.10 | -0.27-0.07 | 0.238 |
| IELSG* (per 1 point increase) |  | -0.13 | -0.27-0.02 | 0.088 |

**Supplementary Table 4. Univariable predictors of 1^st^-line progression-free survival (PFS) and overall survival (OS) in patients with PCNSL (n=48).** All data are from univariable Cox proportional hazards regression models. *Clinical stage was not analysed as only 1 patient had stage III/IV disease. Abbreviations: PFS – Progression-free survival, OS – Overall survival, HR – Hazard ratio, 95%CI – 95% confidence interval, p – Wald test p-value, BMI – Body Mass Index, ECOG – Eastern Cooperative Oncology Group performance status, N/A – not applicable, DLBCL – Diffuse Large B-cell lymphoma, DEL – double expressor lymphoma, IPI – International Prognostic Index, R-IPI – Revised International Prognostic Index, NCCN-IPI – National Comprehensive Cancer Network International Prognostic Index, MSKCC – Memorial Sloan Kettering Cancer Centre risk score for PCNSL outcomes, IELSG – International Extranodal Lymphoma Study Group risk score for PCNSL outcomes (without cerebrospinal fluid).

| **ENDPOINT** |  | **PFS (Progression-free Survival)** | | |  | **Overall Survival (OS)** | | |
| --- | --- | --- | --- | --- | --- | --- | --- | --- |
|  |  |  |  |  |  |  |  |  |
|  |  | **HR** | **95%CI** | **p** |  | **HR** | **95%CI** | **p** |
| **Demographics** |  |  |  |  |  |  |  |  |
| Female gender |  | 0.86 | 0.41-1.82 | 0.700 |  | 0.85 | 0.40-1.84 | 0.687 |
| BMI at diagnosis (per 5kg/m² increase) |  | 1.01 | 0.60-1.71 | 0.970 |  | 1.06 | 0.62-1.82 | 0.828 |
| Age at diagnosis (per 5 years increase) |  | 1.19 | 1.01-1.40 | 0.035 |  | 1.26 | 1.06-1.49 | 0.009 |
| ECOG (per 1 point increase) |  | 1.26 | 0.87-1.83 | 0.216 |  | 1.32 | 0.90-1.94 | 0.151 |
|  |  |  |  |  |  |  |  |  |
| **Tumour characteristics** |  |  |  |  |  |  |  |  |
| Clinical stage III/IV* |  | N/A | N/A | N/A |  | N/A | N/A | N/A |
| DLBCL “Cell of origin” Germinal centre (GC) |  | 0.79 | 0.19-3.34 | 0.750 |  | 0.69 | 0.16-2.93 | 0.619 |
| DEL biology (per 1 DEL feature increase) |  | 3.19 | 1.87-5.46 | <0.0001 |  | 3.26 | 1.86-5.72 | <0.0001 |
| ---DEL biology: 0 DEL features |  | Ref. | Ref. | Ref. |  | Ref. | Ref. | Ref. |
| ---DEL biology: 1 DEL feature |  | 5.02 | 1.38-18.29 | 0.014 |  | 4.16 | 1.13-15.32 | 0.032 |
| ---DEL biology: 2 DEL features |  | 12.81 | 3.48-47.15 | <0.0001 |  | 10.72 | 2.83-40.61 | <0.0001 |
| Involvement of deep brain structures |  | 0.61 | 0.30-1.23 | 0.165 |  | 0.60 | 0.29-1.22 | 0.158 |
|  |  |  |  |  |  |  |  |  |
| **Risk stratification systems** |  |  |  |  |  |  |  |  |
| IPI (per 1 point increase) |  | 1.38 | 0.95-2.02 | 0.092 |  | 1.57 | 1.07-2.30 | 0.021 |
| R-IPI (per 1 point increase) |  | 1.38 | 0.95-2.02 | 0.092 |  | 1.57 | 1.07-2.30 | 0.021 |
| NCCN-IPI (per 1 point increase) |  | 1.49 | 1.05-2.12 | 0.024 |  | 1.73 | 1.21-2.49 | 0.003 |
| MSKCC (per 1 point increase) |  | 1.58 | 0.92-2.70 | 0.094 |  | 1.88 | 1.05-3.36 | 0.033 |
| IELSG* (per 1 point increase) |  | 1.17 | 0.78-1.75 | 0.449 |  | 1.28 | 0.85-1.93 | 0.245 |

**Supplementary Table 5. Two multivariable models of DEL biology and the NCCN-IPI for 1^st^-line progression-free survival (PFS) and overall survival (OS) in patients with PCNSL (n=48).** All data are from multivariable Cox proportional hazards regression models. Abbreviations: 95%CI – 95% confidence interval, p – Wald test p-value, PFS – Progression-free survival, OS – Overall survival, DEL – double expressor lymphoma, NCCN-IPI – National Comprehensive Cancer Network International Prognostic Index. Ref. – Reference category.

| **Variable** |  | **Multivariable Hazard Ratio** | **95%CI** | **p** |
| --- | --- | --- | --- | --- |
|  |  |  |  |  |
| **Multivariable Model #1:**  **Progression-free Survival (PFS)** |  |  |  |  |
| DEL biology: 0 DEL features |  | Ref. | Ref. | Ref. |
| DEL biology: 1 DEL feature |  | 4.31 | 1.16-16.02 | 0.029 |
| DEL biology: 2 DEL features |  | 10.90 | 2.92-40.76 | <0.0001 |
| NCCN-IPI (per 1 point increase) |  | 1.26 | 0.88-1.81 | 0.207 |
|  |  |  |  |  |
| **Multivariable Model #2:**  **Overall Survival (OS)** |  |  |  |  |
| DEL biology: 0 DEL features |  | Ref. | Ref. | Ref. |
| DEL biology: 1 DEL feature |  | 4.16 | 1.13-15.32 | 0.032 |
| DEL biology: 2 DEL features |  | 10.72 | 2.83-40.61 | <0.0001 |
| NCCN-IPI (per 1 point increase) |  | 1.53 | 1.05-2.24 | 0.027 |
